# Biomarker Gene Signature Discovery Integrating Network Knowledge

**DOI:** 10.3390/biology1010005

**Published:** 2012-02-27

**Authors:** Yupeng Cun, Holger Fröhlich

**Affiliations:** Bonn-Aachen International Center for IT (B-IT), Dahlmannstr. 2, 53113 Bonn, Germany; Email: cun@bit.uni-bonn.de

**Keywords:** biomarker, gene selection, protein-protein interactions network, personalized medicine

## Abstract

Discovery of prognostic and diagnostic biomarker gene signatures for diseases, such as cancer, is seen as a major step towards a better personalized medicine. During the last decade various methods, mainly coming from the machine learning or statistical domain, have been proposed for that purpose. However, one important obstacle for making gene signatures a standard tool in clinical diagnosis is the typical low reproducibility of these signatures combined with the difficulty to achieve a clear biological interpretation. For that purpose in the last years there has been a growing interest in approaches that try to integrate information from molecular interaction networks. Here we review the current state of research in this field by giving an overview about so-far proposed approaches.

## 1. Introduction

In recent years, the topic “personalized medicine” has gained much attention. A famous example is the anticancer drug Cetuximab, which binds to the EGF receptor and, consequently, prevents activation of the downstream signaling pathway, thus inhibiting cell proliferation. However, it has been found that Cetuximab can work only if the K-RAS gene is not mutated. Testing patients for mutations of this gene in the European Union is thus prescribed before application of Cetuximab to prevent a costly and ultimately ineffective therapy. Another example is the anti-cancer drug Trastuzumab, which is only effective in patients that express highly the human epidermal growth factor (HER2) at the cell surface, to which the antibody binds.

These examples underline the need for identifying reliable biomarkers that predict a patient’s response to therapy, including potential adverse effects, in order to avoid ineffective treatment and to reduce drug side-effects and associated costs. Towards that goal a large amount of work has been conducted within the last decade, which tries to stratify patients according to disease subtypes or different clinical prognosis. Nowadays modern high-throughput technologies allow for screening of massive amounts of OMICs-type data, and so one goal is to associate such data with a patient’s clinical prognosis or with the membership to a certain disease subtype. Based on OMICs data it has been even possible to identify novel disease subtypes. For example, based on gene expression profiles, five subtypes of breast cancer have been identified [1].

Prognostic or diagnostic biomarker signatures (mostly from gene expression data, but more recently also from other data types, such as miRNA, methylation patterns or copy number alterations) have been derived in numerous publications for various disease entities. One of the best known ones is a 70-gene signature for breast cancer prognosis (mammaprint) by van ’t Veer *et al.* [2], which has gained FDA approval.

For the construction of biomarker signatures, one typically uses supervised machine learning methods together with algorithms for variable/feature selection. This is because OMICs data is typically very high dimensional compared to the number of samples/patients in a typical study. The microarray technology nowadays enables measurement of tens of thousands of transcripts at the same time, whereas the sample size is typically in the order of 100–300 patients. This not only imposes high challenges for the interpretation of such data, but also for robust and stable statistical procedures, which are needed to detect those genes that are truly correlated with the clinical phenotype. In this context it should be mentioned that typical machine learning algorithms operating with far more variables/features than samples are prone to the so-called “overfitting” phenomenon: The classifier or Cox regressor can perfectly explain the data used for model construction, but fails in making good predictions on new test data [3,4]. Therefore algorithms and statistical procedures for efficient reduction and selection of relevant features of the data are crucial.

Well known algorithms for this purpose are PAM [5], SVM-RFE [6], Random Forests [7] or statistical tests, like SAM [8], in combination with conventional machine learning methods (e.g., Support Vector Machines, k-NN, LDA, logistic regression,...). An excellent overview about these algorithms can be found in Hastie *et al.* [4]. Moreover, several modifications of Support Vector Machines (SVMs) for embedding gene selection into this algorithm have been proposed [9,10,11]. For associating gene expression or other high-dimensional experimental or clinical data with patient survival times, typically Cox regression or variations thereof (multivariate penalized Cox regression) are employed [12,13]. However, retrieved gene signatures are often not reproducible in the sense that inclusion or exclusion of a few patients can lead to quite different sets of selected genes. Moreover, these sets are often difficult to interpret in a biological way [14]. For that reason, more recently a number of approaches have been proposed, which try to integrate knowledge on canonical pathways or protein-protein interactions into gene selection algorithms. The general hope is not only to make biomarker signatures more stable, but also more interpretable in a biological sense. This is seen as a key to making gene signatures a standard tool in clinical diagnosis [15]. In this review we would like to give an overview about these approaches and highlight their specific features.

## 2. Techniques

### 2.1. Overview

Nowadays knowledge on protein-protein interactions (PPIs) as well as on canonical pathways can be retrieved easily in a computer readable format from databases, such as KEGG [16], HPRD [17], PathwayCommons [18] or others. These databases contain collections of protein interactions that have been reported in the literature. What has to be mentioned, however, is that usually these interactions have been observed under differing biological conditions and cell types. Thus a purely literature based network reconstruction will suffer from a lack of specificity with respect to the cell or tissue type under study. Moreover, false interactions can be frequently observed due to technological limitations, which are, for instance, imposed by genome scale two-hybrid or co-precipitation screens. Hence, confidence measures for interactions are of high value [19,20]. On the other hand it is widely believed that only a fraction of the true interactome is known so far. Despite these limitations network reconstructions have turned out to provide valuable hypotheses for biomarker signature discovery. 

In general one may divide existing methods integrating network knowledge broadly into two main classes: On one hand there are network centric approaches, which map gene expression data onto a PPI network reconstructed from the literature and then either try to identify discriminative/differential sub-networks between patient groups, or directly compute summary statistics (pathway activity) for pre-defined sub-networks (e.g., canonical pathways). Afterwards often a conventional classifier (e.g., logistic regression, k-NN) or Cox regressor is applied to make predictions based on the expression profiles of sub-network genes. 

On the other hand data centric approaches are closer to traditional machine learning methods. Here the idea is to bias the gene selection process within a machine learning framework in such a way that connected genes are preferably selected. There are two main techniques for this purpose: One is to construct a mathematical embedding of gene expression data into a network graph space via the so-called kernel trick [21]. Afterwards existing kernel-based feature selection algorithms, such as SVM-RFE [6], can be applied. Another approach is to modify the feature selection process itself, e.g., by imposing specific restrictions on the learnable parameters (so-called regularization) [22]. 

In the following we give a more detailed overview about these methods. 

### 2.2. Network Centric Approaches

#### 2.2.1. Network Features

An approach, which is possibly most focusing on the network structure itself, is to purely select genes based on topological features of the protein-protein interaction network. An example is the method proposed in Taylor *et al.* [23]. Here the idea is to concentrate on hubs in the network, *i.e.*, proteins with an extraordinary high degree of interactions. In their paper Taylor *et al*. show that the average Pearson correlation of the expression of a hub protein and its interacting partners can be used to reliably predict survival of breast cancer patients without any further machine learning based variable or feature selection procedure. 

#### 2.2.2. Pathway Activity

Another method to integrate network knowledge is to summarize the expression level of predefined canonical pathways obtained from databases, such as KEGG [16], into one value, for instance by taking the mean or the median. These newly constructed interpretable features are then correlated with the clinical phenotype to be predicted using conventional machine learning techniques. 

Guo *et al.* [24] report that “functional expression profiles” obtained by taking the average expression of genes annotated to significantly enriched Gene Ontology (GO, The Gene Ontology Consortium [25]) categories could increase the robustness of a classifier trained to discriminate four cancer types. 

Rather than simply looking at mean or median expression Vaske *et al.* [26] propose a probabilistic approach based on a factor graph model for pathway activity inference from both gene expression and copy number alterations. In contrast to many others, this method is completely probabilistic and takes the topology of the pathway into account. 

Teschendorff *et al.* [27] further decompose pathways into coherent modules based on the correlation structure in gene expression data. For each module an activation metric is proposed, which specifically takes into account the network architecture. Penalized Cox regression is then used to associate pathway activity to survival times of ER-positive breast cancer patients. 

Another approach following the same direction is proposed by Trey Ideker and co-workers [28]. In their paper an activity score is derived from the normalized expression of most discriminative genes within each pathway. Logistic regression is applied to discriminate between “good” or “bad” prognosis breast cancer patients based on these scores. In their paper Lee *et al*. show that their “combined optimal response genes” (CORGs) approach yields better prediction performance than if pathway activity is simply estimated via the mean or median expression level. A further improvement of the method with respect to the selection of discriminative genes within each pathway is proposed in Yang *et al.* [29]. 

Bild *et al.* [30] estimate pathway activity by so-called “meta-genes”, which are obtained by computing the first principal components of the expression of pathway genes. The authors use their method to cluster several tumor entities and identify coordinated patterns of pathway deregulation, which distinguish between specific cancers and tumor subtypes. Bild *et al*. show that estimated pathway activities are predictive for the respective patient subgroups, and that in cell lines pathway activity also predicts the sensitivity to therapeutic compounds. An extension of the pathway activity classifier to identify oncogene-inducible modules is described in Bentink *et al.* [31]. 

Yu *et al.* [32] propose to first detect pathways that are significantly associated with the phenotype via a global test strategy [33]. Afterwards genes annotated to these pathways are selected based on their individual differential expression. Using their approach the authors successfully establish an interpretable signature for predicting metastasis of lymph node negative breast cancer patients. 

The paper by Kammers *et al.* [34] focuses on functional gene groups defined by GO. Rather than computing an explicit measure of group activity, the authors first identify group representatives via PAM clustering [35]. Penalized Cox regression is then used to associate expression of group representatives as well as several clinical covariates to survival times of breast cancer patients. 

#### 2.2.3. Differential Sub-Networks

Rather than looking at predefined canonical pathways or GO groups, another idea that puts a little bit more emphasis on measured data is to reconstruct a protein-protein interaction network for all gene products and then use experimental data to identify differentially expressed sub-networks. One of the first approaches in this direction is described in Chuang *et al.* [36]. The algorithm starts from “seed” proteins in the network, which are highly differentially expressed. Then around each seed protein neighbors are added in a greedy hill climbing fashion until the discriminative power of the corresponding sub­network (measured via the mutual information of the average normalized gene expression together with the clinical outcome variable) reaches a local maximum. In their paper Trey Ideker and co-workers show that their method not only leads to clearly interpretable signatures for discriminating “poor” and “bad” prognosis breast cancer patients, but also improves prediction performance compared to a conventional machine learning setup. Similar greedy algorithms for identification of differential sub-networks have been proposed by other authors, e.g., Chowdhury and Koyutürk [37], Fortney *et al.* [38], Su *et al.* [39], Ahn *et al.* [40]. 

A particular interesting variant has recently been introduced by Dutkowski and Ideker [41]. They modify Random Forests [42], which contain a large ensemble of decision trees, such that individual trees only use neighboring genes in the PPI network. This allows them to draw conclusions about the inherent logic by which stably selected sub-networks are dis-regulated. The authors show that their method leads to a much better reproducibility of selected markers compared to using a conventional Random Forest. 

It has to be mentioned that despite their good performances, all so far mentioned approaches are heuristic and thus cannot guarantee to find the *optimal* differential sub-network. Attempts to obtain an optimal sub-network are described in Chowdhury *et al.* [43] via branch and bound and in Dao *et al.* [44] via exhaustive search. A particular elegant solution is proposed by Dittrich *et al.* [45]. After calculating a score for differential expression of each node in the protein-protein interaction network, the authors interpret the problem of identifying the optimal differential sub-network as an instance of the prize-collecting Steiner tree problem, which they solve to optimality via integer linear programming (ILP). The authors show that their obtained optimal sub-networks generally correlate well with the clinical phenotype of diffuse large B-cell lymphomas, however no rigorous validation in terms of prediction accuracy is performed. 

In general, identification of an optimally discriminative sub-network is an NP-hard problem [45,46] and thus algorithms have to face a super-polynomial run time complexity, which can make them intractable for larger datasets. An interesting compromise between computational speed and the goal to obtain a well separating sub-network has thus recently been proposed in Dao *et al.* [46]. Their algorithm is based on the color coding paradigm [47], which allows for identifying optimally discriminative sub-networks up to a certain error rate. Dao *et al*. use a randomized approximation algorithm to obtain polynomial run time complexity. Afterwards the authors employ a 3-NN classifier on averaged expression levels of each sub-network to discriminate response to chemotherapy in breast cancer. 

### 2.3. Data Centric Approaches

#### 2.3.1. Mathematical Embedding

All previously mentioned approaches deal with a PPI network as the central entity. In contrast, data centric approaches focus on the experimental data. Kernel techniques [21] allow for a mathematically elegant way of combining network information with experimental data. Kernels play a crucial role for Support Vector Machine classifiers. 

In general, a kernel function *k* : 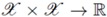 can be thought of as a special similarity measure between arbitrary objects *x* ∈ 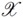, which fulfills additional mathematical requirements, namely symmetry (*i.e.*, *k*(*x*,*y*) = *k*(*y*,*x*) for all *x*,*y* ∈ 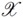) and positive semi-definiteness (*i.e.*, *k*(*x*,*y*) = 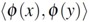 for all *x*,*y*, where (·) denotes the dot product in Hilbert space 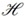 and 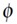: 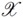 → 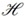 is some arbitrary map) [21,48]. Among many other applications kernel functions have been proposed for nodes in a graph or network based on the notion of random walks. A random walk is a stochastic process that consists of a sequence of moves that are taken along the graph structure according to some defined probability distribution. The diffusion kernels [49] is a specific similarity measure for nodes in a graph that considers all random walk paths connecting nodes *x* and *y*, but weights each path in dependency on the path length. This is done in an exponentially decreasing way. Diffusion kernels are mathematically equivalent to the fundamental solution of the heat equation in physics, which describes the evolution of heat in a region under certain boundary conditions. If instead of exponentially decreasing weights for path lengths a linear weighting scheme is preferred, one arrives at the pseudo-inverse of the graph Laplacian [50]. In the same paper also a random walk kernel is proposed, which simply bounds the number of random walk steps to *p*. 

The aforementioned graph kernels allow for easily incorporating measurement data, such as gene expression. This is done by weighting each edge *x* → *y* in the network by the similarity of the gene expression of *x* and *y* (using the dot product). This is equivalent to defining a kernel function between *x* and *y* as:

*k*(*x*,*y*) = **x***^T^*** Qy**

where **x** and **y** are the vectors of gene expression values for genes *x* and *y*, and **Q** is the graph kernel matrix between nodes in the network. Consequently the expression data is linearly mapped via the graph kernel matrix Q to some different space. 

Combining gene expression data with network information in such a way has been described by Rapaport *et al.* [51] and Gao *et al.* [50]. In general the intuition of these methods is that genes which are closely connected in the network should also have similar expression levels. Rapaport *et al*. in particular emphasize the possibility to conduct unsupervised clustering analysis of gene expression data in this way besides more common supervised classification, which yields biologically interpretable results. Several other authors have used graph kernels to identify possibly disease causing genes [52,53]. 

Recently, Chen *et al.* [54] have introduced a variation of the kernel idea using the pseudo-inverse of the graph Laplacian. In their paper the authors compute an explicit mapping of gene expression data by a matrix square root of Q, which is calculated via singular value decomposition. An ordinary linear Support Vector Machine is then trained on the transformed data. Afterwards the solution is back-transformed to the original space and a permutation test executed for assessing the significance of genes and identifying sub-networks. With their approach the authors successfully predict early *vs.* late recurrence of ER positive breast cancer patients with comparably high accuracy. Moreover, the obtained sub-network markers appear to be biologically plausible. 

#### 2.3.2. Biased Feature Selection

Instead of augmenting the similarity measure of each pair of genes with network information via embedding techniques, another approach is to directly integrate network information into conventional variable/feature selection techniques. Zhu *et al.* [55] describe a modified Support Vector Machine (SVM) algorithm with embedded feature selection, which strongly prefers to select genes that are connected to each other. Via their method the authors successfully obtain sub-networks associated to Parkinson’s disease and to breast cancer metastasis. 

Johannes *et al.* [56] introduce a modification of the frequently used SVM-RFE algorithm, called SVM-RRFE (Reweighted Recursive Feature Elimination). They use the GeneRank approach [57], which is based on Google’s famous PageRank algorithm [58] to identify genes that on one hand exhibit a high fold change and on the other hand are central in the PPI network. With this ranking they re-adjust the SVM decision hyperplane, which is learned at each step of the SVM-RFE algorithm. This way they give preference to selecting genes, which have a high GeneRank. It can be shown that this approach is equivalent to run the conventional SVM-RFE algorithm on data that is transformed in a specific way, *i.e.*, embedded into a different space. In their paper the authors demonstrate that SVM-RRFE is not only superior to the conventional SVM-RFE algorithm in predicting an early relapse in breast cancer patients, but can also compete with several other network based gene selection approaches. Moreover, the stability and interpretability of the obtained gene signatures are significantly improved. 

Binder and Schumacher [13] propose a component-wise likelihood boosting approach for integrating network information. The idea is to decrease the penalty for selecting variables/genes that are connected in the PPI network. The authors demonstrate on two gene expression datasets, diffuse large B-cell lymphoma and ovarian cancer, that their approach is able to improve survival time predictions via a multivariate penalized Cox regression model compared to conventional likelihood boosting for the same purpose. 

In a recent paper Gade *et al.* [20] extend the method by Binder and Schumacher [13] by considering a miRNA-mRNA interaction graph rather than a PPI network. Gade *et al*. show that this way miRNA and mRNA expression data can be combined in a straightforward way for predicting the risk of a relapse in prostate cancer via penalized Cox regression. Moreover, they demonstrate that their approach enhances prediction performance and gene selection stability compared to several other methods. 

Lasso regression models [59] have gained a particular attention for high dimensional data analysis during the last years. Li and Li [60] propose a modification of this approach, which down-weights the penalty for selecting genes that are in proximity to each other. They demonstrate that their method can improve over the conventional lasso for predicting survival of glioblastoma patients. Despite the elegance of the approach it has to be mentioned that the authors do not consider the possible censoring of patient survival times in their study. Hence, the application of a conventional regression framework in this context has to be considered critical. 

## 3. Discussion and Conclusions

Integration of biological knowledge, specifically from protein-protein interaction networks and canonical pathways, is widely accepted as an important step to make biomarker signature discovery from high dimensional data more robust, stable and interpretable. Consequently there is an increasing amount of methodologies for this purpose. In this review we gave a general overview about these approaches and grouped them into categories. 

The majority of algorithms at the moment follow the network centric paradigm, specifically by looking for differentially expressed sub-networks. This approach certainly appears attractive, because the gene selection problem is solved in a very elegant and natural way. Moreover, learned sub-networks are usually better to interpret than gene signature obtained via conventional machine learning techniques. One difficulty, however, lies in the fact that usually sub-networks are found by connecting differentially expressed genes. If there are no differentially expressed genes, then the identification of discriminative sub-networks becomes difficult. Moreover, finding an optimally discriminative sub-network is a computationally difficult, NP-hard problem. 

Estimating the total activity of canonical pathways appears to be comparably simpler from a computational point of view. However, the approach only works if many genes in a pathway change their expression in a coordinated way, *i.e.*, the phenotype can be explained by the activity of defined canonical pathways. If this is not the case or if only a few genes within a pathway are slightly differential, then identifying a pathway’s activity to be associated to the clinical phenotype becomes difficult. Hence, smart filtering strategies to focus on the most relevant genes within a pathway, like in the CORGs algorithm [28] or via the method described in Yu *et al.* [32], are useful. However, even then the problem remains that not all phenotypes might be explainable by the activities of defined canonical pathways. 

There are comparably few data centric approaches, which come from the machine learning field, specifically from the area of kernel based methods. Since OMICs data is very high dimensional, linear classifiers are usually sufficient and thus the biased feature selection framework appears to be more natural. Compared to network centric approaches, these methods have the potential advantage that they do not rely so much on the PPI network structure. That means false positives or false negative interactions in the network structure will affect the model less. In addition, they are able to detect sub-networks, which are not part of canonical pathways. Thus they can be seen as more generally applicable. On the other hand they use available biological knowledge in a much less effective way than network centric methods. 

In conclusion we see that all approaches that have been proposed so far have specific advantages and disadvantages. Thus there is a strong need for systematic empirical comparisons. Cun and Fröhlich [61] conducted a comparison of 14 classification algorithms (8 using network knowledge) for predicting early *vs.* late relapse of breast cancer patients in 6 microarray datasets. They found a large variability of prediction performances of individual algorithms across datasets, but no general advantage of network based methods. Network based SVM approach by Zhu *et al.* [55] yielded the most stable signatures, but only revealed a comparably low prediction performance, whereas RRFE signatures [56] had the highest enrichment in terms of disease related genes, pathways and known drug targets. Average pathway expression in this study was found to lead to clearly interpretable signatures coupled with a good prediction performance. 

In this context it has to be emphasized that most published methods have been evaluated for one specific clinical question (e.g., early relapse prediction) in one disease (mostly breast cancer), only. To get a more complete picture, more comprehensive studies including more clinical questions and more disease entities are needed in order to guide practitioners, under which conditions which method would be a good choice. Nonetheless, there will be always a dataset specific dependency of an algorithm’s performance, which can never be resolved. Careful checking of assumptions is therefore a prerequisite for the successful application of any algorithm. 
